# Low-Frequency Sound-Insulation Performance of Labyrinth-Type Helmholtz and Thin-Film Compound Acoustic Metamaterial

**DOI:** 10.3390/ma17184475

**Published:** 2024-09-12

**Authors:** Peizhou Hu, Jingbo Zhao, Hong Liu, Xiaosheng Zhang, Guangjun Zhang, Hong Yao

**Affiliations:** Air Force Engineering University, Xi’an 710000, China; hu496804307@163.com (P.H.); lhconquer@163.com (H.L.); peanut1015@163.com (X.Z.); zhanggj3@126.com (G.Z.); yyaohong@hotmail.com (H.Y.)

**Keywords:** acoustic metamaterial, sound insulation, Helmholtz cavity, low frequency, labyrinth type, sound-insulation peak

## Abstract

This paper presents a type of acoustic metamaterial that combines a labyrinth channel with a Helmholtz cavity and a thin film. The labyrinth-opening design and thin-film combination contribute to the metamaterial’s exceptional sound-insulation performance. After comprehensive research, it is observed that in the frequency range of 20–1200 Hz, this acoustic metamaterial exhibits multiple sound-insulation peaks, showing a high overall sound-insulation quality. Specifically, the first sound-insulation peak is 26.3 Hz, with a bandwidth of 13 Hz and giving a transmission loss of 56.5 dB, showing excellent low-frequency sound-insulation performance. To further understand the low-frequency sound-insulation mechanism, this paper uses the equivalent model method to conduct an acoustic–electrical analogy, construct an equivalent model of the acoustic metamaterial, and delve into the sound-insulation mechanism at the first sound-insulation peak. To confirm the validity of the theoretical calculations, physical experiments are carried out by 3D printing experimental samples. The analysis of the experimental data has yielded results that are consistent with the simulation data, providing empirical evidence for the accuracy of the theoretical model. The material has significant practical application value. Finally, various factors are studied in depth based on the established equivalent model, which can provide valuable insights for the design and practical engineering application of acoustic metamaterials.

## 1. Introduction

The issue of noise pollution has consistently proven to be a major challenge affecting the daily lives of individuals. There is a lot of evidence that when people live in a noisy environment for a long time, they will be irreversibly damaged physically and psychologically, which places a huge burden on people’s physical and mental health [1,2,3]. Therefore, it is essential to reduce noise pollution. In everyday settings, the frequencies associated with noise pollution are large, which can be mainly divided into high-frequency bands and low-frequency bands. Traditional sound-insulation and noise-reduction materials can effectively solve the high-frequency band noise pollution; however, due to the low-frequency noise band, where the sound wave is long, traditional materials usually need a large volume to produce an effect, which makes the cost rise rapidly and, at the same time, makes it difficult to obtain satisfactory results. However, it is difficult to put into practical applications, so seeking a kind of acoustic metamaterial or superstructure with light mass, small volume, low cost, and high efficiency is the best way to solve the low-frequency noise in daily life.

Acoustic metamaterials [4,5,6,7,8,9] are new synthetic materials that have been artificially designed and are usually designed with subwavelength composite structures. Due to their special structural units, they can exhibit novel acoustic properties different from traditional materials, such as a negative Poisson ratio, a negative mass density, and a negative equivalent bulk modulus, by carefully designing the constituent materials and structures [10,11,12,13,14,15]. In 2000, Liu [16] initiated the development of a local resonant phonon crystal, marking a milestone in the field of acoustic metamaterials that has since attracted substantial interest from the global academic community. Based on local resonance theory, acoustic metamaterials such as thin-film metamaterials have been designed by taking advantage of their low-frequency characteristics [17,18]. Mixed-coil-type acoustic metamaterials [19], perforated-plate-type acoustic metamaterials [20], Helmholtz-cavity-type acoustic metamaterials [21], and other different types of acoustic metamaterials can realize the function of “small size control large wavelength”, which has strongly promoted the development of acoustic metamaterials. Bucciarelli [22] presents a multiple-layer MPP absorber with a high sound-absorption coefficient and broadband absorption at low frequencies, which can guarantee constant high absorption of over 90% in the frequency range of 400 to 2000 Hz. Lu [23] designed a novel membrane-based acoustic metamaterial that has high transmission loss at low frequencies and studied its mechanism using negative mass density theory. Brahim Lemkalli [24] proposed a novel lightweight acoustic metamaterial panel composed of coupled Helmholtz resonators that can effectively isolate low-frequency broadband noise. Yang [25] pioneered the development of a tunable Helmholtz shunt acoustic metamaterial, incorporating a procedure that introduces multiple resonant chambers to expand the absorption bandwidth of each chamber and manipulate the tuning length of the rear cavity. This innovative design was designed to facilitate the procurement of a wide range of absorption vocal cords.

However, acoustic metamaterials with a single structure will have their own flaw. Helmholtz cavity acoustic metamaterials can play a role at low frequencies, but the frequency range of the role is small, and it is difficult to achieve a large range of control. Thin-film acoustic metamaterials are faced with problems such as difficult tension control and easy aging of the material, which affect the stability of acoustic performance. Compound acoustic metamaterials with multiple design structures have been proposed. Li [26] designed a new sandwich structure whose average sound-insulation performance at 50–1600 Hz is 30% higher than that of the single-film sandwich structure, and the sound-insulation performance is 24.8% higher than that of the double-film structure without increasing the chamber height. He [27] cleverly devised an acoustic metamaterial using the principle of a Helmholtz cavity in conjunction with a thin-film structure, which has good sound-insulation performance in the frequency range of 20–1200 Hz. Hu [28] proposed an innovative acoustic metamaterial design that incorporated a series of membrane-coupled Helmholtz resonators to induce multiple band gaps in the low-frequency range, thereby facilitating comprehensive broadband noise reduction.

The studies proposed in this paper are important for the design and implementation of acoustic metamaterials. However, there is still a lack of research on compound acoustic metamaterials in low-frequency sound insulation, and they have limited practical application value. This paper introduces a Helmholtz cavity with labyrinth opening and thin-film compound acoustic metamaterial and uses the finite element method to evaluate the performance of the system over the 20–1200 Hz frequency range. For sound-insulation performance, multiple resonance modes were examined, the resonance mechanism was elucidated, and an equivalent model was devised, with theoretical calculations validated by real-world experiments. Using an equivalent-circuit model, the effects of multiple parameters on the sound-insulation efficiency of metamaterials were investigated. The results show that the proposed model can produce a noise isolation peak in the low-frequency system, in addition to exhibiting a substantial transmission loss, suggesting its potential for practical application and engineering relevance. This metamaterial can be widely used in aircraft, submarines, vehicles, and other devices. Due to the influence of engines, propellers, and other components, it can cause huge noise pollution and has great health hazards and operating effects on operators. Due to the advantages of small mass and good performance, the metamaterial can be widely used in the inner wall of the device. For example, the helicopter can use the material to build the inner cabin wall, which can not only effectively reduce weight but also better solve noise pollution.

## 2. Structure Design and Calculation Method

### 2.1. Structural Design

**Figure 1:**
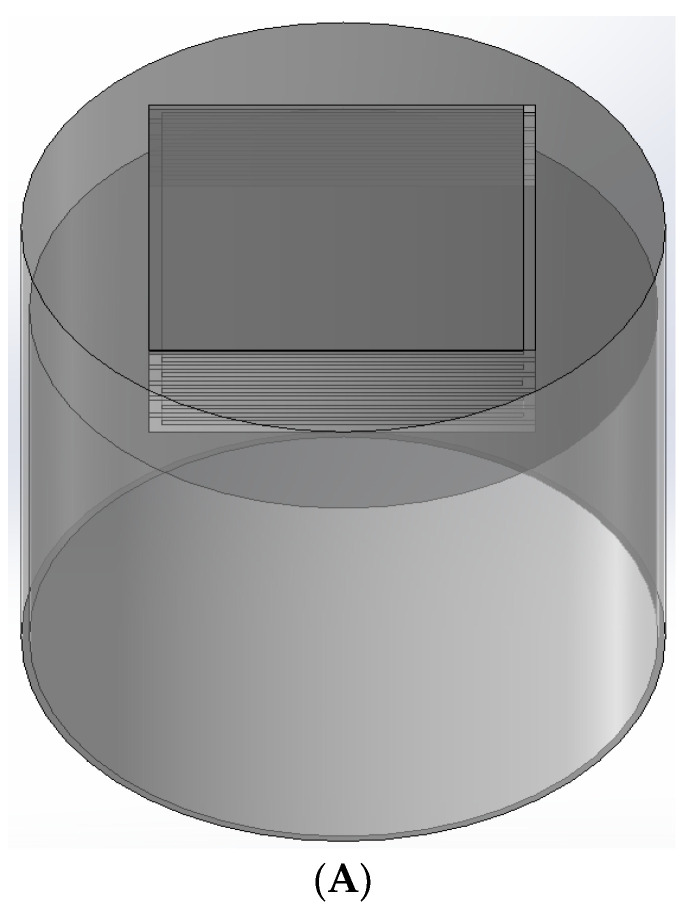

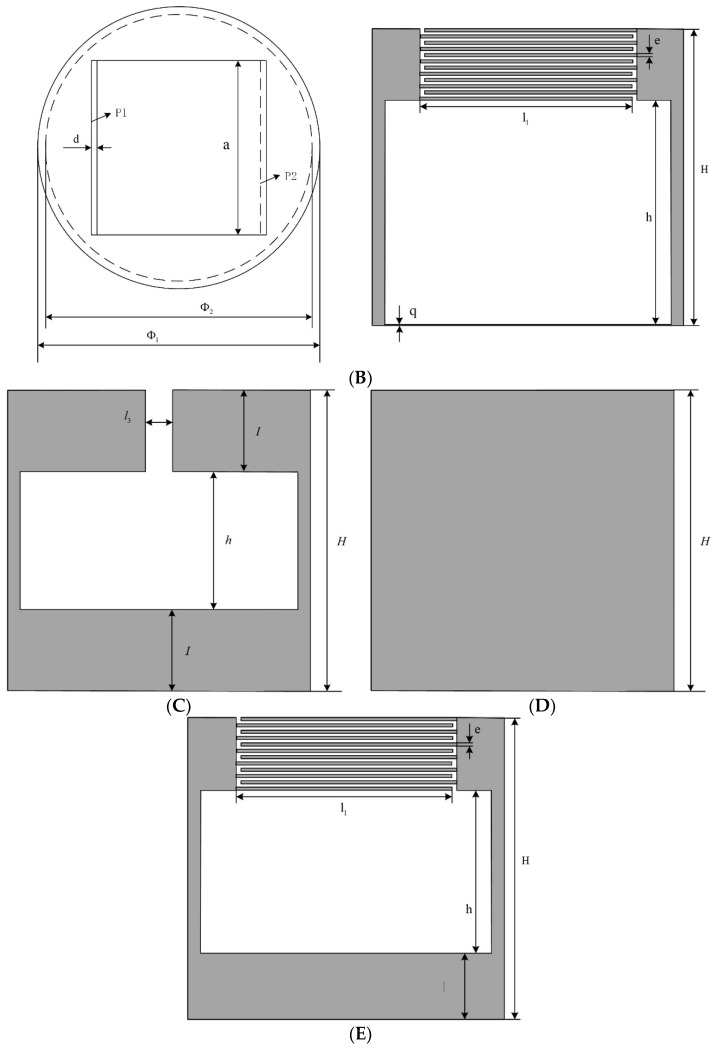
(**A**) Structure diagram; (**B**) parameter diagram; (**C**) single Helmholtz cavity; (**D**) traditional solid material; (**E**) single-opening labyrinth.

The labyrinth-type Helmholtz and thin-film composite acoustic metamaterial structure designed in this paper is shown in Figure 1, where P1 and P2 are both open channels. The material is a cylindrical structure with a cylindrical cavity inside, a circular film at the lower part, and a labyrinth opening channel at the upper part. It is composed of n = 10 plates arranged and combined in an orderly manner, wherein the outer diameter $\Phi_{1}$ = 100 mm, the inner diameter $\Phi_{2}$ = 92 mm, the plate length a = 65 mm, the narrow slit width d = 2 mm, the outer cylinder height H = 100 mm and the inner cylinder height h = 80 mm, the plate thickness e = 1 mm, the plate width $l_{1}$ = 64 mm, the film thickness q = 0.8 mm, and the tension on the film f=0.4 MPa. The labyrinth opening design takes full advantage of the available space, amplifying the length of air passage, enhancing the propagation distance of sound waves, and decreasing the peak frequency of sound insulation, ultimately improving the sound-insulation performance. In addition, the Helmholtz cavity structure can provide superior sound-insulation performance in the low-frequency domain. When integrated with the labyrinth opening, the material can exhibit a low-frequency sound-insulation peak. A thin-film design is implemented, allowing the material to exhibit exceptional sound-insulation performance in the low-frequency band and to produce multiple sound-insulation peaks, thereby enhancing the overall sound-insulation performance. The structures of the single Helmholtz cavity, the solid cylinder structure, and the single-opening labyrinth type are shown in Figure 1C–E, where $l_{3}$ = 10 mm and I = 27 mm, and the shaded part is the solid part. In the simulation, the material used is photosensitive resin. 

### 2.2. Numerical Model

To calculate the sound-insulation characteristics of metamaterials, a finite element method simulation was carried out using the sound pressure coupling module of COMSOL Multiphysics 6.1 software. In the simulation, the physical field of thermoviscous acoustics was added, rubber was used as the film material, and solid steel was used as the remaining material. Table 1 provides the detailed material parameters used in the experiment. Due to the substantial difference in acoustic impedance between rigid steel and air, sound waves are expected to have a difficult time passing through solid steel. Therefore, for simplicity of calculation, we can assume that sound waves will only have a minor impact when passing through the structural frame, and the corresponding loss can be safely ignored. In this paper, the structural frame is treated as a fixed constraint in the simulation, thus ignoring any influence caused by the vibration of the frame.

**Figure 2:**
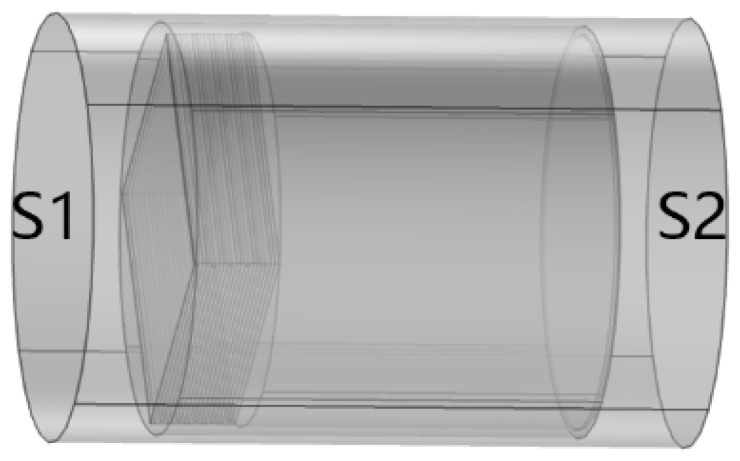
The model diagram used in the simulation process (S1 is the incident end, S2 is the exit end).

In COMSOL, the finite element method was used to model and calculate the transmission loss TL of metamaterials, as shown in Figure 2. Here, the air field was represented by the outer cylinder, and the perfect sound-absorbing layer was set on the left and right sides. The left, S1, was defined as the plane-wave incident port, and the right, S2, was defined as the plane-wave exit port. The incident wave excitation is set at the incident port. Due to the simplicity of the simulation calculation, the incident wave power is defined as 1 W for the simulation. To ensure that the full area of the metamaterial is contained within the air domain, a diameter of 101 mm for the outer cylinder was selected, which slightly exceeds the diameter of the metamaterial. When dividing the grid, the default general setting can divide the grid size to meet the simulation requirements, so the grid size setting directly adopts the default setting.

In the calculation process, the transmission loss TL is defined as follows:

(1) $$\left\{\begin{array}{c} W_{in}=\int_{s_{1}}\frac{p_{i}^{2}}{2\rho c}dS\\ W_{out}=\int_{s_{2}}\frac{p_{t}^{2}}{2\rho c}dS\\ TL=10\log(\frac{W_{in}}{W_{out}}) \end{array}\right.$$

where $p_{i}$ represents the incident sound pressure of S1, $p_{t}$ represents the transmitted sound pressure through S2, ρ represents the air density, c represents the propagation speed of sound in the air, $W_{\mathrm{in}}$ represents the incident sound energy, and $W_{\mathrm{out}}$ represents the outgoing sound energy.

## 3. Analysis of Sound-Insulation Mechanism and Establishment of Equivalent Model

### 3.1. Analysis of Sound-Insulation Mechanism

**Figure 3:**
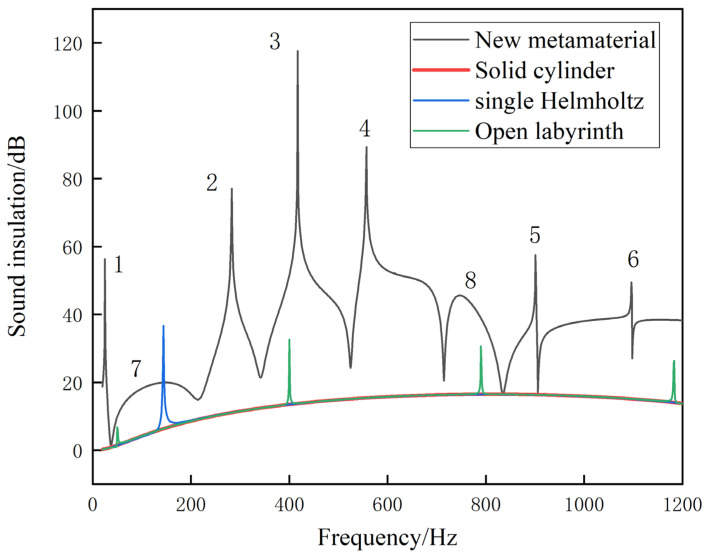
Transmission loss curves for multiple models.

As shown in Figure 3, the simulation results show that the proposed metamaterial exhibits multiple high-bandwidth sound-insulation peaks in the range of 20 to 1200 Hz. This feature suggests that the sound-insulation performance of metamaterials is outstanding. The first peak is 26.3 Hz, with a bandwidth of 13 Hz and a transmission loss of 56.5 dB. The second peak is 283.5 Hz, where the transmission loss reaches 77.3 dB. The third peak is 417.1 Hz, corresponding to a transmission loss of 117.8 dB. The fourth peak is detected at 557.2 Hz, with a transmission loss of 88.9 dB. The fifth and sixth peaks are found in the high-frequency range above 800 Hz, which has not yet been studied. Despite the absence of significant sound-insulation peaks in the seventh and eighth bands, they still exhibit high transmission losses and wide frequency ranges. It should be noted that, compared to traditional Helmholtz cavities, solid cylinders, and open labyrinth structures, the proposed metamaterial significantly outperforms them in terms of transmission loss and frequency band. The simulation was carried out using resin as the material, and the film uses rubber as a material.

Based on the illustration, it is apparent that the profile of the solid cylinder is significantly overlapped by the profile of the single Helmholtz cavity structure and the single labyrinth opening structure. Conventional vibration isolation and noise reduction materials are mainly solid substances, lacking a sound-insulation peak on the transmission loss curve, and while they are heavy, their results are subpar. Although the traditional Helmholtz structure has a lower quality and better effect, it usually only has peak resonance sound insulation, and the sound-insulation band is narrow, resulting in an overall effect that is less than ideal. The single-opening labyrinth structure outperforms the single Helmholtz cavity structure; it may have lower frequency and higher sound-insulation peaks, but its overall performance is still less than satisfactory. The acoustic metamaterial proposed in this paper has multiple broadband sound-insulation peaks, and it still maintains high sound insulation at low frequency, demonstrating an overall performance that is satisfactory. In conclusion, based on the evidence provided, the proposed acoustic metamaterials exhibit superior overall performance.

**Figure 4:**
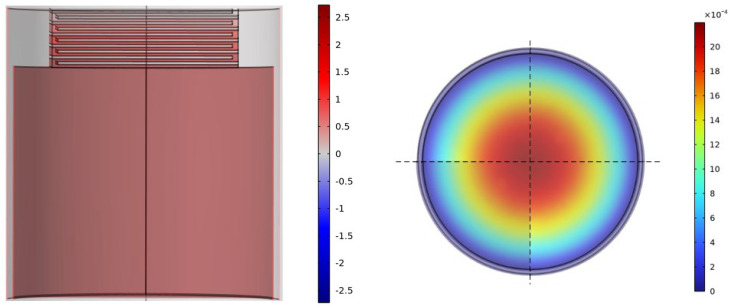
First sound−insulation peak frequency modal diagram.

The pattern diagram corresponding to the frequency of the first sound-insulation peak is shown in Figure 4. Analysis of the sound pressure pattern diagram and vibration pattern diagram at this peak shows that, at the frequency of the first sound-insulation peak, the resonance mode of the metamaterial will be excited due to the proximity of this frequency to its resonance frequency, and coupling resonance will occur between the thin-film structure and the air in the cavity. For the sound pressure pattern diagram or the part of the cavity and labyrinth channel where metamaterials act, sound pressure occurs mainly within the cavity and at specific labyrinth channels. When exposed to the incident wave, the air in the labyrinth channel will experience strong vibration. At this time, the air inside the cavity will behave like a spring and exert an elastic force on the air in the opening hole. This process may result in the energy of the incident wave being consumed in the labyrinth channel and vibrating air friction with the hole wall; the effect of this phenomenon is to absorb a proportion of the energy of the incident wave. In addition, the labyrinth channel air will radiate sound waves outward as it vibrates, causing energy dissipation. Regarding the pattern diagram, the thin-film structure will also experience strong resonance. During this phase, the thin-film structure exhibits a first-order resonance at the frequency in question, and the vibration of the tensioner film is in a symmetrical state, which effectively localizes the energy of the incident wave within the structure and dissipates it. Therefore, the sound-insulation performance of the structure is particularly enhanced.

### 3.2. Equivalent Modeling

The material’s first low-frequency sound-insulation peak represents its outstanding low-frequency sound-insulation capability, which is a key feature of this product. Based on the above analysis of the resonance mode of the material, it is clear that the resonance frequency of the material greatly influences its sound-insulation performance. Specifically, the first-order resonance frequency, which is the corresponding frequency of the first peak, essentially determines a low-frequency peak of material. To provide a more complete understanding of the material’s sound-insulation mechanism, an equivalent model has been created, which can quickly calculate the first low-frequency sound-insulation peak. This is crucial for assessing the material’s low-frequency sound-insulation capability.

**Figure 5:**
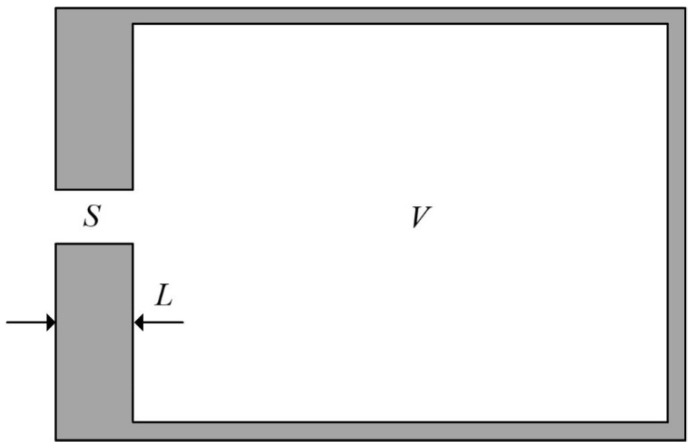
Helmholtz cavity structure simplified diagram.

For the conventional Helmholtz cavity structure, as shown in Figure 5, its structure can be regarded as a rigid body structure; the cross-sectional area is S, the length is L, and the volume in the cavity is V.

Where the port of the tube is subjected to sound waves of sound pressure equal to $p=p_{a}e^{j\omega t}$, the Helmholtz resonator may be considered an acoustic vibration system of spring–vibrator structure. The gas in the short tube acts as the vibrator, and the gas in the cavity acts as the spring. Within this acoustic vibration system, the vibration equation is

(2) $$\left\{\begin{array}{c} M_{a}\frac{dU}{dt}+R_{a}U+\frac{1}{C_{a}}\int Udt=p_{a}e^{j\omega t}\\ U=vS \end{array}\right.$$

where $M_{a}$ represents the mass of sound, $R_{a}$ represents the resistance of sound, $C_{a}$ represents the capacity of sound, v is the air velocity, and U is the velocity in the volume.

**Figure 6:**
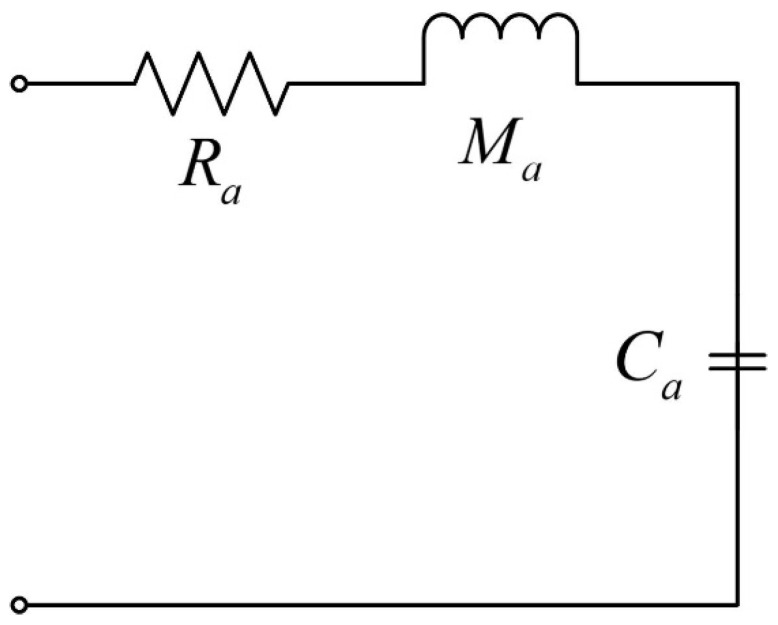
Helmholtz cavity equivalent-circuit diagram.

Due to the similarity of the differential equations and their solutions to the circuit oscillation system, mechanical vibration system, and acoustic vibration system, an electrical–force–acoustic analogy can be made. Therefore, the traditional Helmholtz cavity structure can be transformed into the equivalent-circuit structure shown in Figure 6, where the acoustic resistance is $R_{a}$, the sound quality is $M_{a}$, and the sound sensation is $C_{a}$.

The resonant frequency $f_{a}$ of the structure is

(3) $$f_{a}=\frac{1}{2\pi }\sqrt{\frac{1}{M_{a}C_{a}}}$$

For the general planar film, its motion equation can be obtained by the element method. Assuming that the film is in the xy plane at equilibrium, the tension in the film T is equal and constant everywhere, and η is the vertical displacement of a point on the film from the equilibrium position, the vibration equation of the film can be obtained as follows:

(4) $$\nabla^{2}\eta =\frac{\sigma }{T}\cdot \frac{\partial^{2}\eta }{\partial t^{2}}$$

where σ is the mass of the film per unit area, and $\nabla^{2}=\frac{\partial^{2}}{\partial x^{2}}+\frac{\partial^{2}}{\partial y^{2}}$ is the Laplacian operator of the two-dimensional cartesian coordinates.

In this paper, the metamaterial is a symmetrical, freely vibrating circular film with a fixed perimeter. The physical condition of the circular film with a fixed perimeter can be mathematically reduced to finding the grounding of the zero-solution column Bessel function $J_{0}(z)$, whose first-order resonance frequency is

(5) $$f_{1}=\frac{\mu_{1}}{2\pi r}\sqrt{\frac{T}{\sigma }}$$

where r is the circumferential radius of the circular film, and $\mu_{1}=2.045$ is a specific value, which is a specific value satisfying the solution to the cylindrical Bessel function.

**Figure 7:**
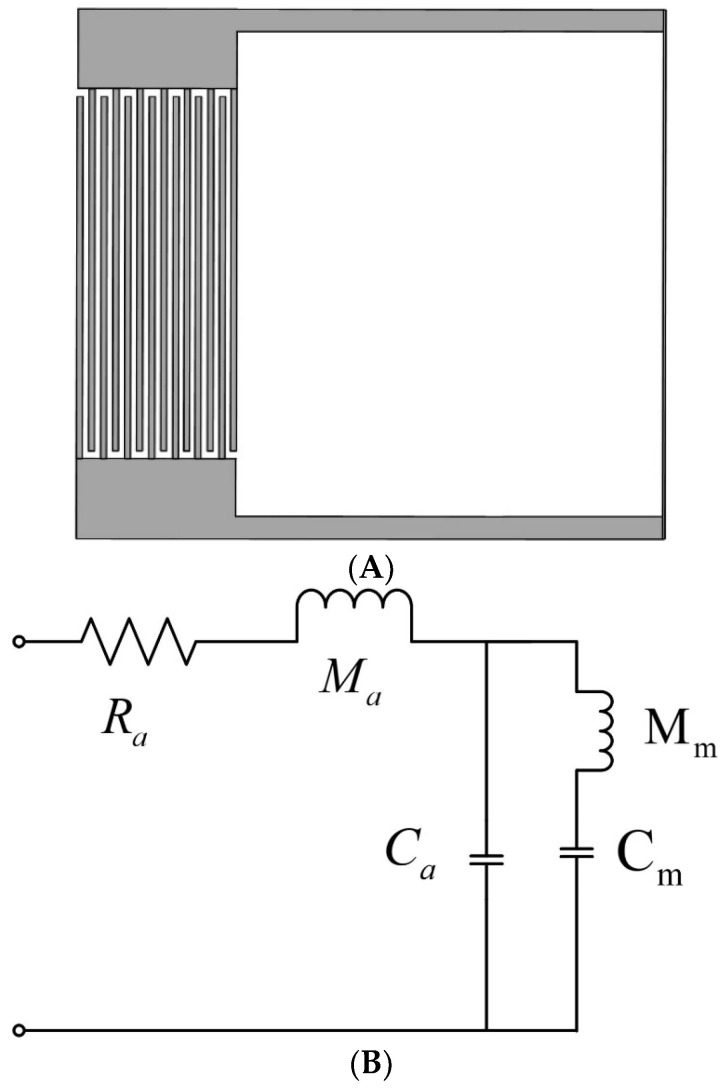
(**A**) Metamaterial structure diagram; (**B**) metamaterial structure equivalent-circuit diagram.

For the metamaterial structure proposed in this paper, the labyrinth passage and its intracavitary components can be approximated as Helmholtz cavities since the bottom is composed of a thin film made of rubber, the bottom cannot be considered a rigid structure, while the rest can still be considered a rigid body, and the equivalent-circuit diagram at this time is shown in Figure 7.

From the above analysis, it can be seen that the first resonance mode of the film is symmetrical vibration, which can be equivalent to the “spring–vibrator” system for analysis:

(6) $$\left\{\begin{array}{c} K_{f}=\omega^{2}mJ_{1}^{2}(\mu )\\ M_{f}=mJ_{1}^{2}(\mu )\\ \omega =2\pi f=\sqrt{K_{f}/M_{f}} \end{array}\right.$$

where $M_{f}$ represents the equivalent mass, $K_{f}$ represents the equivalent stiffness, m represents the film mass, and ω represents the circular frequency.

Assuming that the pressure on the film is uniform and the film area is $S_{m}$, the equivalent coefficient of the force–sound converter is $A=13/S_{m}$ because the surrounding boundaries are fixed:

(7) $$\left\{\begin{array}{c} C_{m}=\frac{S_{m}^{2}}{9\omega^{2}mJ_{1}^{2}(\mu )}\\ M_{m}=\frac{9mJ_{1}^{2}(\mu )}{S_{m}^{2}} \end{array}\right.$$

Then, the total impedance corresponding to the equivalent circuit of the structure is

(8) $$Z=R_{a}+j\omega M_{a}-j\frac{\omega M_{m}-1}{\omega^{3}M_{m}C_{a}C_{m}-\omega C_{a}-\omega C_{m}}$$

When $Z=R_{a}$, f corresponding to solving the circuit equation is the resonance frequency.

Comparative data on the results and associated numerical errors achieved through the equivalent model methodology and finite element method are summarized in Table 2. Here, the numerical error is calculated using the finite element method as the true value.

## 4. Experimental Verification

To validate the accuracy of the equivalent model and the numerical calculation, an experimental approach was adopted using real-life objects. Recently, with the development of 3D printing technology, it has become possible to create experimental samples through 3D printing. First, a physical model was fabricated by 3D printing, and then an acoustic insulation measurement of the AWA6290T transfer function was performed. Next, transmission loss detection experiments were carried out on the real model in the frequency range of 50–1000 Hz. Experimental data were carefully recorded and evaluated alongside simulation results.

**Figure 8:**
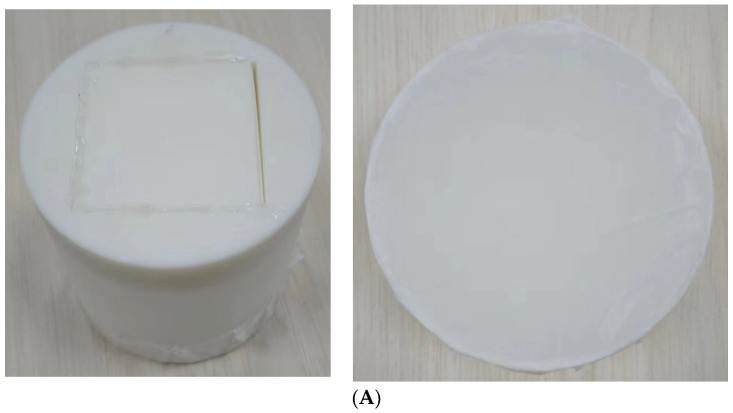

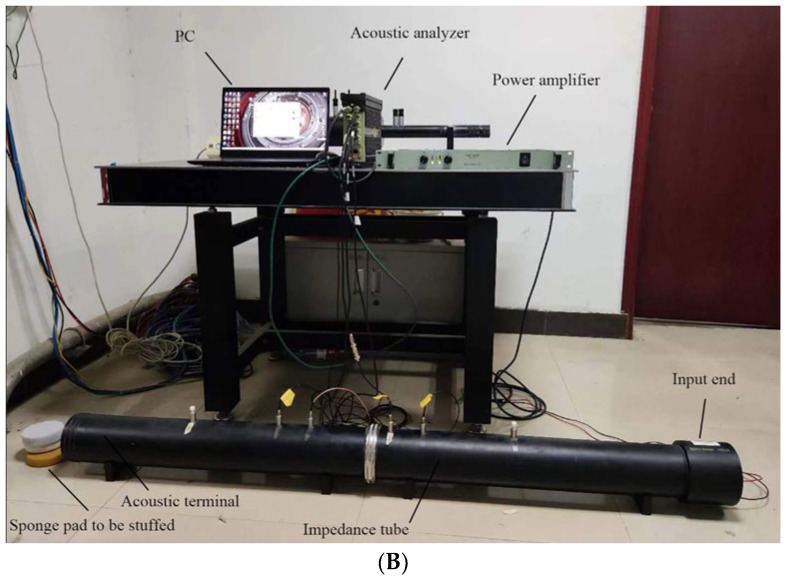
(**A**) Machining sample for experiment; (**B**) experimental equipment and experimental process diagram.

Given that the primary mechanism underlying the sound insulation of the metamaterial is derived from its unique structure and is not materially dependent, nor does it consider the influence of vibrational energy within the structural framework itself, it can be assimilated to a rigid body in terms of sound-insulation characteristics. Drawing on the lightweight properties of metamaterials, during the simulation experiment, the 3D printing process uses resin as the material. The acoustic impedance of air is set to 0.0004 Rayleigh, and the acoustic impedance of the resin is 1.8 Rayleigh. Compared to the acoustic impedance of resin, the impact of the acoustic impedance of air is negligible, so it can be ignored. Therefore, it can be assumed that using resin for printing test samples does not significantly affect the sound performance. In the actual process of the physical sample, the selection of sample parameter values is consistent with the structural design parameter values mentioned in Section 2. To enhance the accuracy of the experimental measurement and minimize the impact of errors, it is recommended that six 10 cm diameter sponges be affixed to the rear end of the impedance tube during the test phase. The experimental setup is illustrated in Figure 8. The impedance tube is divided into two sections: the input end and the sound absorption end. The test sample is positioned within the impedance tube, and a sponge pad is positioned at the sound absorption end to absorb sound waves. The white noise generated by the data acquisition front end is transmitted to the speaker via the power amplifier, and the sound pressure signal in the pipeline is captured by the sensor. The acquired data are then transferred to the acoustic analyzer, where they are analyzed and calculated by the software on the computer to yield the experimental data.

**Figure 9:**
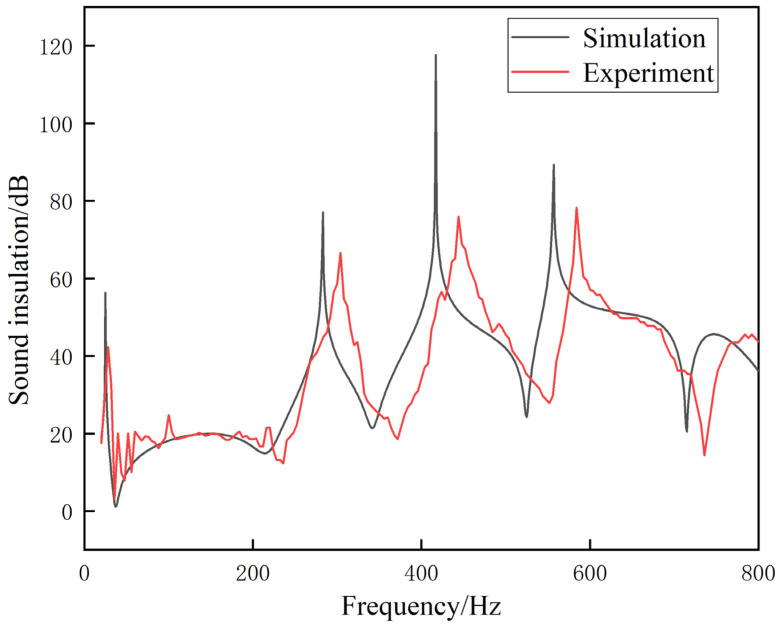
Comparison of experimental transmission loss data and simulation results.

As shown in Figure 9, despite the observed discrepancies between the physical experimental results and the simulation results, the curve trends remain consistent, indicating a level of alignment between the overall data. As such, it can be considered that the experimental results are reliable, and the numerical results are consistent with the actual results, which validates the theoretical calculation. The primary causes of any discrepancies between the numerical computation and physical experimentation can be attributed to the following: (1) The experimental samples were produced using a multistage printing splicing process and adhered to glue, which may have altered the actual volume of the inner cavity and air passage. (2) The dimensions of the air passage and aperture are relatively minute, leading to substantial deviations in actual print production, and the accuracy of the printing model parameters is substandard [29,30]. (3) The minor system error identified in the measurement system is difficult to correct but does not significantly affect transmission loss. (4) The surface tension of the film cannot be precisely controlled, causing the sound-insulation peaks measured by the experiment and the simulation peaks to differ. (5) The complex labyrinth channel configuration results in narrow gaps between the plates, resulting in significant viscous and thermal interaction between air and solid material within the channel. This complex process induces significant dampening effects, which significantly affect acoustic performance.

## 5. Exploring the Determinants of Performance Evaluation of Low-Frequency Sound Insulation

In the Section 3, a rigorous equivalent-circuit model of metamaterials is proposed and validated by a thorough comparison with the results obtained from the finite element method. Using this equivalent model, the factors influencing the first low-frequency resonance frequency of metamaterials are discussed. This shows that the first resonance frequency of metamaterials is representative of metamaterial properties, thus highlighting the importance of studying this frequency. By analyzing the model, it is revealed that the film thickness, the film tension, the labyrinth channel length, and the inner cavity volume are the main factors affecting metamaterials. Using the variable control method, this study carries out simulation experiments in COMSOL Multiphysics 6.1 software to study the effect of these four factors on the first peak frequency sound insulation of metamaterials. The finite element method results are recorded, and the sound-insulation mechanism is further elucidated.

### 5.1. Effect of Film Thickness on the Frequency of the First Sound-Insulation Peak

**Figure 10:**
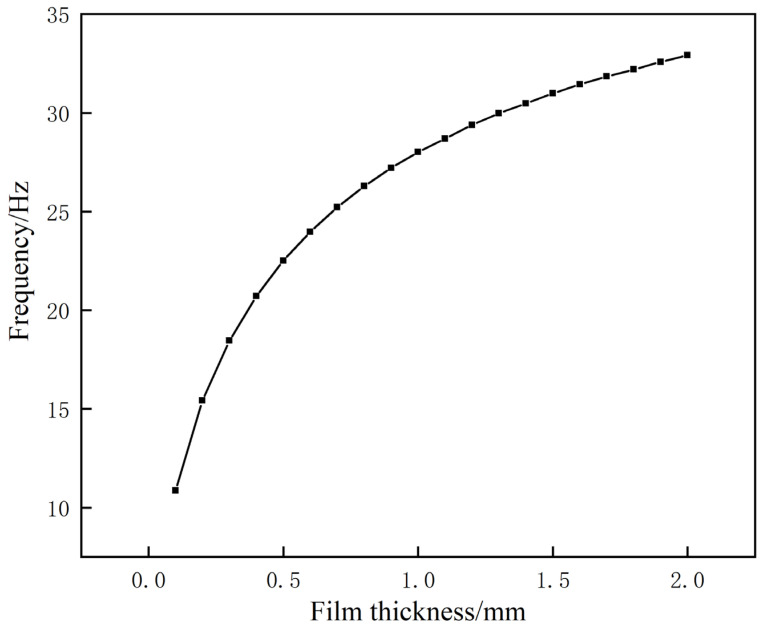
Examination of the effect of film thickness on the frequency of the initial sound-insulation peak.

Based on the control variable method, when studying the effect of the film thickness on low-frequency sound insulation, the other parameters were kept unchanged, and the film thickness was changed. The thickness was increased from 0.1 mm at the beginning to 2.0 mm in increments of 0.1 mm. The frequency change in the first sound-insulation peak is shown in Figure 10.

After reviewing the data, we observed that the resonance frequency of the first sound-insulation peak consistently increases with an increase in film thickness. This observation can be attributed to the increased film mass resulting from the increased film thickness. According to the established equivalent model formula, when other parameters remain constant, an increase in film mass will invariably lead to an increase in resonance frequency. This confirmation is consistent with the results of the simulation experiment and leads us to the conclusion that for the structure, the higher the film thickness, the higher the frequency of the first sound-insulation peak.

### 5.2. Effect of Film Tension on the Frequency of the First Sound-Insulation Peak

**Figure 11:**
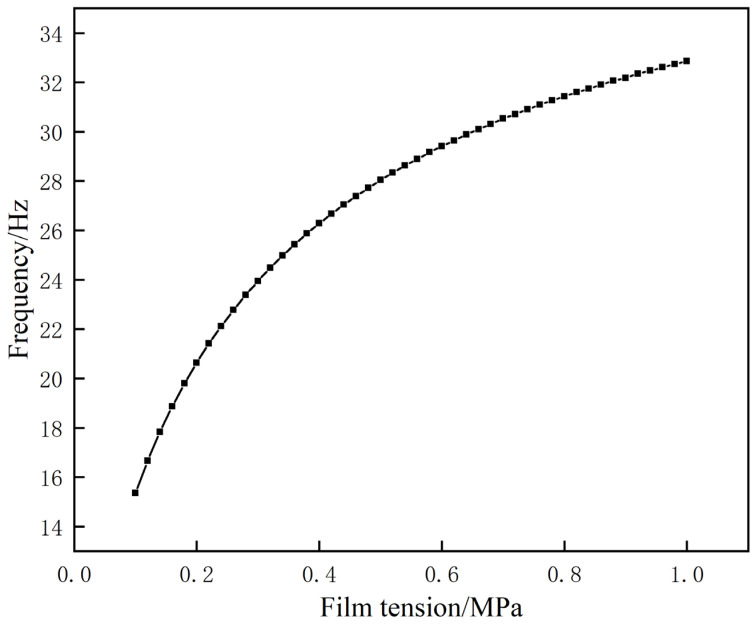
Effect of film tension on the frequency of the first sound-insulation peak.

During course of the above simulation experiment, all other relevant parameters were maintained unaltered. Instead, the film tension was incrementally increased from a value of 0.1 MPa to a final value of 1 MPa, with a step increment of 0.02 MPa. The frequency change curve of the first sound-insulation peak was obtained, as shown in Figure 11.

After careful examination of the data provided, it is clear that as the film tension increases, the peak frequency of primary sound insulation steadily increases. This observation is primarily driven by the correlation between tension and film surface tightness. As tension increases, the film surface tightness increases, which in turn contributes to the increase in the fundamental positive frequency of the film section calculated using the zero-order Bessel curve. As a result, the calculated resonance frequency increases as the tension increases. Nevertheless, it is crucial to point out that the tension setting of metamaterials is subject to specific limitations. The tension value should not be increased indefinitely, rather it should be confined to a specific range. Moreover, as the tension increases, the risk of damage to metamaterials also increases. Therefore, the tension value should be determined according to the specific application and should not be set too high.

### 5.3. The Influence of the Initial Orientation on the Amplitude of the Initial Sound-Insulation Peak

**Figure 12:**
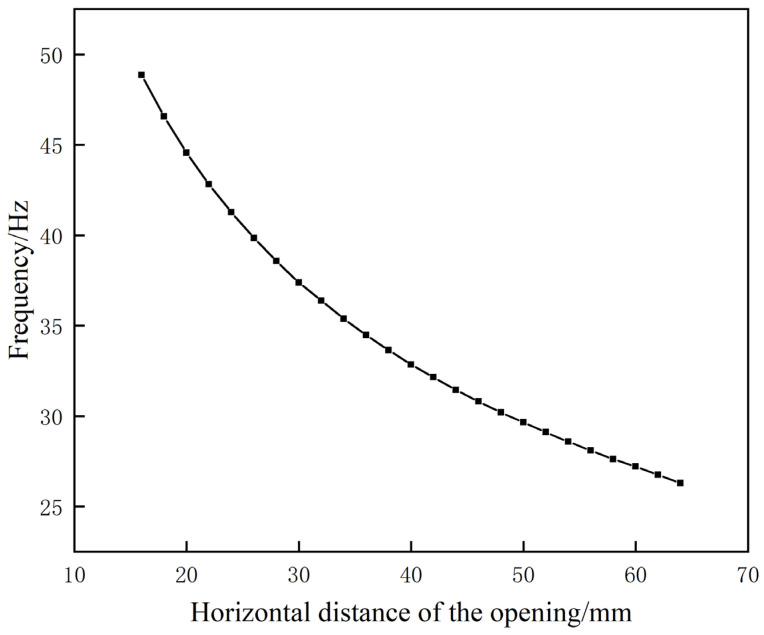
The influence of the opening position on the frequency of the initial sound-insulation peak.

Using a variable control methodology, this study will investigate the impact of aperture location on the performance of low-frequency sound insulation by maintaining the stability of all other experimental variables. Specifically, the horizontal distance between two apertures will be modified by reducing the distance from the initial value of 64 mm to 16 mm, with a step size of 2 mm. The frequency change in the first sound-insulation peak is shown in Figure 12.

The graph shows that as the horizontal distance from the opening position decreases, the resonant frequency associated with the first sound-insulation peak continues to decrease. The condition of the artifact’s improved air passage length on both sides, resulting in a change in distance, is attributed to this phenomenon. The established equivalent model predicts that a decrease in the equivalent inductance of the air passage, with all other parameters constant, will lead to a reduction in the resonant frequency. The correlation established between the equivalent model and the simulation experiment validates the model. Accordingly, it can be determined that for this particular structure, the first resonance frequency is appreciably lower when the horizontal distance between the two openings is increased.

### 5.4. Effect of Cavity Volume on the Frequency of the First Sound-Insulation Peak

**Figure 13:**
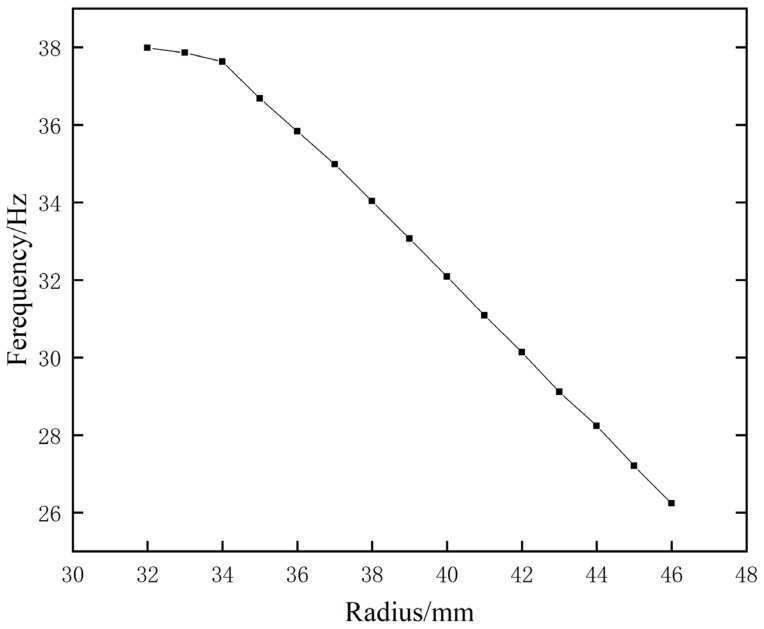
Effect of cavity volume on the frequency of the first sound-insulation peak.

In the context of this simulation experiment, all other parameters were maintained in their original settings, with the exception of the internal volume, which was variably altered by continuously adjusting the radius of the cylinder constituting the internal cavity. The radius was reduced from 46 mm at the beginning to 32 mm in increments of 1 mm. The frequency change curve of the first sound-insulation peak was obtained, as shown in Figure 13.

After careful examination of the data provided, it is apparent that an increase in the dimensions of the inner cavity invariably manifests itself as a corresponding decrease in the frequency of the initial sound-insulation peak, thereby enhancing the overall sound transmittance capacity. Conversely, as the dimensions of the inner cavity decrease, the frequency of the initial sound-insulation peak increases, resulting in a reduction in the overall sound-insulation effectiveness. This correlation is mainly due to the formulated equivalent model, where a larger inner cavity is correlated with a smaller equivalent capacitance and thus a lower frequency of the initial sound-insulation peak. However, the inner cavity is subject to certain physical constraints, as the radius cannot be increased indefinitely. Therefore, the diameter of the inner cavity must be strategically designed according to the specific conditions.

## 6. Conclusions

In this paper, a kind of acoustic metamaterial with a single bilateral opening Helmholtz cavity is designed:

(1) Through the use of finite element analysis, the sound-insulation performance of metamaterials over a frequency range of 20–1200 Hz was comprehensively evaluated. This analysis identified several notable peaks in sound-insulation performance, reflecting the overall high efficiency of sound insulation. A particularly notable feature was the presence of a sound-insulation peak at a minimum frequency of 26.3 Hz, indicative of the material’s robust sound-insulation capability. These experimental results were fully consistent with the theoretical calculations, providing validation of the theoretical model’s accuracy and further supporting the use of metamaterials as potential sound-insulation materials.
(2) In order to better understand the sound-insulation mechanism of metamaterials, a comprehensive analysis of sound pressure and vibration patterns at the frequency of the first sound-insulation peak was carried out. The equivalent model method was used to construct the corresponding model. The consistency between the results of the equivalent model method and those of the finite element method was used to verify the accuracy of the equivalent model and gain further insights into the sound-insulation mechanism.
(3) The equivalent model established in (2) was used to investigate the factors that influence the sound insulation of metamaterials. The results of the analysis revealed that increasing the horizontal distance between two openings and the volume of the inner cavity can substantially reduce the first resonance frequency, thereby enhancing the sound-insulation performance of metamaterials. In addition, it should be noted that the larger the aperture width and the farther the aperture position is from the central plane, the higher the first resonant frequency will be. Therefore, specific parameter settings should be determined based on the specific application scenario.

## Figures and Tables

**Table 1:** Material parameters.

Materials	Poisson’s Ratio	Density (kg/m^3^)	Young’s Modulus (Pa)
Rubber	0.469	1300	$1.175\times 10^{5}$
Resin	0.340	1100	$4.32\times 10^{6}$

**Table 2:** Comparison of calculation results.

Method	First Peak Frequency of Sound Insulation
Equivalence modeling approach	25.9 (Hz)
Finite element method	26.3 (Hz)
Inaccuracies	1.29%

## Data Availability

The data that support the findings of this study are available from the corresponding author, upon reasonable request.
